# Draft genome sequences of *Mycolicibacterium peregrinum* isolated from a pig with lymphadenitis and from soil on the same Japanese pig farm

**DOI:** 10.1186/s13104-019-4380-3

**Published:** 2019-06-17

**Authors:** Tetsuya Komatsu, Kenji Ohya, Kotaro Sawai, Justice Opare Odoi, Keiko Otsu, Atsushi Ota, Toshihiro Ito, Mikihiko Kawai, Fumito Maruyama

**Affiliations:** 1 Aichi Prefectural Chuo Livestock Hygiene Service Center, 1-306 Jizouno, Miaicho, Okazaki 4440805 Japan; 2 Laboratory of Veterinary Microbiology, Faculty of Applied Biological Sciences, Gifu University, 1-1 Yanagido, Gifu, 5011193 Japan; 3 United Graduate School of Veterinary Sciences, Gifu University, 1-1 Yanagido, Gifu, 5011193 Japan; 4 Education and Research Center for Food Animal Health, Gifu University (GeFAH), 1-1 Yanagido, Gifu, 5011193 Japan; 5 Viral Disease and Epidemiology Research Division, National Institute of Animal Health, National Agriculture Research Organization, 3-1-5 Kannondai, Tsukuba, Ibaraki 3050856 Japan; 6 Gifu Prefectural Chuo Livestock Hygiene Service Center, 1-1 Yanagido, Gifu, 5011112 Japan; 7 Data Science Center, Division of Biological Science, Nara Institute of Science and Technology, Ikoma, Nara 6300192 Japan; 8 Department of Microbiology, Graduate School of Medicine, Kyoto University, Yoshida-Konoe-cho, Sakyo-ku, Kyoto, 6068501 Japan; 9 Scientific and Technological Bioresource Nucleus, Universidad de La Frontera, Av. Francisco Salazar 01145, Temuco, 4811230 Chile; 10 Present Address: Division of Microbiology, National Institute of Health Sciences, 3-25-26 Tonomachi, Kawasaki-ku, Kawasaki, 210-9501 Japan; 11 Present Address: Laboratory of Proteome Research, Proteome Research Center, National Institute of Biomedical Innovation, Ibaraki, Osaka 567-0085 Japan

**Keywords:** *Mycolicibacterium peregrinum*, Draft genome sequence, Mycobacterium, Drug resistance, Virulence, In silico DNA–DNA hybridization, Pig, Pig farm

## Abstract

**Objectives:**

*Mycolicibacterium peregrinum*, a rapidly growing mycobacterial species, can opportunistically infect humans and other animals. Although *M. peregrinum* infections in animals have been reported, the infection sources are unknown, as is information on its virulence and drug resistant genes, which limits our current understanding of this bacterium. To address this knowledge gap, we obtained draft genome sequences for two *M. peregrinum* isolates; one from a case of pig lymphadenitis and one from the pig farm’s soil.

**Data description:**

We report here the draft genome sequences of *M. peregrinum* isolates 131_1 and 138 (6,451,733-bp and 6,479,047-bp). They were isolated from a pig with mesenteric lymph node lymphadenitis and from soil on the Japanese farm where the pig was reared. A sequence alignment identity score of 100% was obtained by in silico DNA–DNA hybridization of the two isolates, while 98.28% (isolate 131_1) and 98.27% (isolate 138) scores were recorded for hybridization with a human isolate. Both isolates carry *arr*-1, *AAC*(2′)-Ib, *RbpA*, *mtrA* and *tap* drug-resistance genes. Isolates 131_1 and 138 carry 234 and 236 putative virulence genes, respectively. Therefore, environment *M. peregrinum* is potentially drug resistant and can cause swine lymphadenitis. Our data provides valuable new information for future studies on nontuberculous mycobacteria.

## Objective

*Mycolicibacterium peregrinum* (basonym: *Mycobacterium peregrinum*), a known pathogenic and rapidly growing mycobacterium (RGM), has been isolated from clinical samples from pigs, cattle and a person [1–3]. Several cases of *M. peregrinum* infection have been reported in aquatic animals [4, 5], wild animals [6–8] and livestock [1, 2, 9], including one porcine case [1]. Nontuberculous mycobacteria (NTM) such as *M. peregrinum* generally reside in water and soil, and these environmental NTM are believed to occasionally infect humans and other species, opportunistically [10]. However, the transmission sources for *M. peregrinum* in humans and other animals are not clear in each case. Classification of the *Mycobacteria* genus currently positions the *Mycobacterium fortuitum* group, including *M. peregrinum*, as *Mycolicibacterium* [11].

Few studies on *M. peregrinum* virulence genes have been conducted [12], but the medical fields have reported on multidrug resistance in this bacterium [13]. It has also been reported that *M. peregrinum* is more susceptible to some antimicrobial agents than other mycobacteria species [14]. Other studies have reported that some RGM carry antibiotic resistance genes, such as erythromycin ribosomal methylase (*erm*) [15], *LfrA* and *tap* [16]. Although the *tap* gene is present in *M. peregrinum*, a comprehensive analysis of its antibiotic resistance genes has not been done. Therefore, to obtain better understanding of the potential risk posed by antibiotic resistance in *M. peregrinum*, an analysis at the draft genome level is necessary. Such information would be useful to veterinary medicine as there is no genome information on isolates from non-human animals. To aid future investigations into the sources of *M. peregrinum* infection and to provide information on virulence and drug resistance genes, we present here the draft *M. peregrinum* genome sequences for isolates 131_1 and 138 from a case of swine lymphadenitis and from soil on the same Japanese farm, respectively.

## Data description

*Mycolicibacterium peregrinum* isolate 131_1 was isolated from the mesenteric lymph nodes of a pig with lymphadenitis and isolate 138 was isolated from soil on the same pig farm (Tokai area of Japan), as described previously [17]. Both samples were individually decontaminated with an equal volume of 2% NaOH and then inoculated onto 2% Ogawa medium (Kyokuto Pharmaceutical, Tokyo, Japan). Both isolates were species identified by sequencing the 16S rRNA, *hsp65* and *rpoB* genes [18, 19]. Genomic DNA was extracted using the PureLink genomic DNA extraction kit (Invitrogen, Carlsbad, CA, USA) according to the manufacturer’s instructions, and paired-end libraries with an average insert size of 350-bp were prepared. Sequencing (2× 150-bp) was conducted on the HiSeq X Ten sequencing platform (Illumina, San Diego, CA, USA) at the Beijing Genomics Institute (Shenzhen, China). Draft genome sequences were obtained from the reads according to the method reported previously (Table 1) [17]. In brief, the reads were trimmed by TrimGalore! (https://github.com/FelixKrueger/TrimGalore) and mismatched reads were corrected, assembled and polished using SPAdes [20], Pilon [21] and Unicycler [22]. Genome completeness was estimated using CheckM [23]. Taxonomic classification was conducted using Kaiju [24] and Anvi’o [25]. Draft genomes were annotated using the NCBI Prokaryotic Genome Annotation Pipeline (PGAP) [26]. Virulence and drug resistant genes were identified by VFanalyser (http://www.mgc.ac.cn/VFs/main.htm) and RGI (https://card.mcmaster.ca/analyze/rgi). In silico DNA–DNA hybridization was conducted by the MUMmer program with JspiecesWS [27].

**Table 1:** Overview of data files

Label	Name of data file/data set	File types (file extension)	Data repository and identifier (DOI or accession number)
Data file 1	*M. peregrinum* 131_1	FASTA file (.fasta)	GenBank RWJZ00000000 (https://www.ncbi.nlm.nih.gov/nuccore/RWJZ00000000)
Data file 2	*M. peregrinum* 138	FASTA file (.fasta)	GenBank RWKA00000000 (https://www.ncbi.nlm.nih.gov/nuccore/RWKA00000000)

The draft genome sequence of *M. peregrinum* isolate 131_1 (Data file 1) comprised 33 contigs with a total length of 6,451,733 bp, a G+C content of 66.41%, and an N50 size of 292,445 bp. The *M. peregrinum* 138 isolate’s draft genome sequence (Data file 2) comprised 46 contigs with a total length of 6,479,047 bp, a G+C content of 66.41%, and an N50 size of 324,444 bp. The coding sequences, rRNAs and tRNAs in both isolates were estimated at 6169, 3, and 55 (isolate 131_1) and 6180, 3, and 55 (isolate 138), respectively. Both isolates contained large numbers of putative virulence genes and genes involved in metabolism (e.g., amino acid, purine, lipid and fatty acid genes), anaerobic respiration, anti-apoptosis, catabolism, metal uptake, cell surface components, mammalian cell entry operons, phagosome arrest, proteases, regulation, secreted proteins, secretion system, stress adaptation and toxins. Both isolates contain five drug resistance-related genes: *arr*-1, *AAC*(2′)-Ib, *RbpA*, *mtrA* and *tap*. In silico DNA–DNA hybridization revealed that the aligned nucleotide sequences from *M. peregrinum* isolates 131_1 and 138 share 98.28% and 98.27% identity with the human *M. peregrinum* isolate [3], respectively, 88.46% sequence identity with *M. fortuitum* subsp. *fortuitum* [28], 85.18% sequence identity with *Mycobacteroides abscessus* [29], 84.60% and 84.61% identity with *M. mucogenicum* [30], respectively, 84.50% sequence identity with *Mycobacteroides chelonae* [31], and 84.21% sequence identity with *M. neoaurum* [32]. An aligned sequence identity score of both isolates was 100%, suggesting that *M. peregrinum* exists in the farm soil and both isolates might possibly be the same origin. Sequencing revealed that both isolates may be resistant to rifampin and macrolide antibiotics. These results provide useful information for future NTM studies and for clinical antibiotic use.

## Limitations

The present data are based on the genome sequences of *M. peregrinum* isolates 131_1 and 138 at the draft level. Therefore, the exact lengths of these sequences, numbers of coding sequences, rRNAs, tRNAs and repetitive elements cannot be predicted with certainty. The existence of plasmid/s or extra-chromosomal DNAs also cannot be predicted with certainty.

## Abbreviations

*LfrA*: the membrane efflux pump gene for quinolones (confers resistance to macrolides)
*erm*: ribosomal RNA methyltransferase gene
*tap*: major facilitator superfamily (MFS) antibiotic efflux pump gene (confers resistance to tetracyclines)
*arr*-*1*: rifampin ADP-ribosyltransferase (Arr) gene
*AAC*(2′)-Ib: chromosomal-encoded aminoglycoside acetyltransferase gene (confers resistance to aminoglycosides)
*RbpA*: RNA-polymerase binding protein gene (confers resistance to rifampin)
*mtrA*: transcriptional activator gene of the MtrCDE multidrug efflux pump (confers resistance to penam, a macrolide antibiotic)

## Availability of data materials

The data described in this Data note can be freely and openly accessed on GenBank. Please see Table 1 and the reference list for details and links to the data accession: RWJZ00000000 (https://www.ncbi.nlm.nih.gov/nuccore/RWJZ00000000) and accession: RWKA00000000 (https://www.ncbi.nlm.nih.gov/nuccore/RWKA00000000).
